# Barriers and facilitators of following perioperative internal medicine recommendations by surgical teams: a sequential, explanatory mixed-methods study

**DOI:** 10.1186/s13741-021-00236-x

**Published:** 2022-02-01

**Authors:** Kristin Flemons, Michael Bosch, Sarah Coakeley, Bushra Muzammal, Rahim Kachra, Shannon M. Ruzycki

**Affiliations:** 1 W21C, University of Calgary, Calgary, Alberta Canada; 2 Department of Medicine, Cumming School of Medicine, University of Calgary, Calgary, Alberta Canada; 3 Cumming School of Medicine, University of Calgary, Calgary, Alberta Canada; 4 Department of Community Health Sciences, Cumming School of Medicine, University of Calgary, Room 1422, Health Sciences Centre, 3330 Hospital Drive NW, Calgary, Alberta T2N 2T9 Canada

**Keywords:** Quality improvement, Perioperative medicine, Internal medicine consultation

## Abstract

**Background:**

Preoperative medical consultations add expense and burden for patients and the impact of these consults on patient outcomes is conflicting. Previous work suggests that 10–40% of preoperative medical consult recommendations are not followed. This limits measurement of the effect of perioperative medical consultation on patient outcomes and represents a quality gap, given the patient time and healthcare cost associated with consultation. We aimed to measure, characterize, and understand reasons for missed recommendations from preoperative medical consultation.

**Methods:**

This explanatory, sequential mixed-methods study used chart audits followed by semi-structured interviews. Chart audit of consecutive patients seen in preoperative medical clinic were reviewed to measure the proportion and characterize the type of recommendations that were not completed (“missed”). This phase informed the interview participants and questions. The interview guide was developed using the Consolidated Framework for Implementation Research and the Theoretical Domains Framework. Template analysis was used to understand drivers and barriers of missed recommendations

**Results:**

Chart audit included 255 patients (*n*=161, 63.1% female) seen in preadmission clinic between April 1 and April 30, 2019. 55.7% of patients had all recommendations followed (*n*=142). Postoperative anticoagulation management and postoperative cardiac biomarker surveillance recommendations were least commonly followed (50.0%, *n*=28, and 68.9%, *n*=82, respectively).

Eighteen surgical team members were interviewed. Missed recommendations were both unintentional and intentional, and the key drivers differed by these categories. Unintentionally missed recommendations occurred due to individual-level factors (drivers: knowledge of the consultation note, lack of routine for reviewing the consultation note, and competing demands on time) and systems-level factors (driver: lack of role clarity). Intentionally missed recommendations occurred due to user error due (drivers: lack of knowledge of guidelines or evidence) and appropriate modifications (driver: need to adapt a preoperative plan for a complicated postoperative course).

**Conclusions:**

Only 55.7% of consult notes had all recommendations followed, suggesting a quality gap in perioperative medical care. Qualitative data suggests multiple drivers of missed recommendations that should be targeted to improve the efficiency of care for these patients.

**Supplementary Information:**

The online version contains supplementary material available at 10.1186/s13741-021-00236-x.

## Background

Preoperative consultation requires an additional appointment for patients and caregivers and adds cost to the surgical process. However, measurement of the benefits of perioperative medical consultation has been challenging. While some studies have demonstrated benefit including reduced mortality (Vazirani et al., 2012), surgical cancellations (Ferschl et al., 2005; Macpherson & Lofgren, 1994), and length of stay (Vazirani et al., 2012), others have reported greater cost (Vazirani et al., 2012; Macpherson & Lofgren, 1994; Auerbach et al., 2007), postoperative complications (Pham et al., 2017; Minai & Kamal, 2004), length of stay (Macpherson & Lofgren, 1994; Auerbach et al., 2007), and no impact on surgical cancellations (Vazirani et al., 2012) or quality of care (Auerbach et al., 2007). A common limitation of these studies is that implementation of perioperative recommendations is not evaluated. Evidence suggests that 15–40% of patients receive no recommendations during a perioperative consultation (Auerbach et al., 2007; Katz et al., 2005) and up to 30% of recommendations are never completed (Katz et al., 2005; Ruzycki et al., 2017). Concurrent evaluation of implementation of perioperative recommendations is needed to accurately measure effect of perioperative consultation.

Further, reasons that these recommendations are not implemented are not well described (Auerbach et al., 2007; Katz et al., 2005). Limited data suggest that recommendations may be unintentionally missed by surgical teams due to communication or systems-level factors (Kluger et al., 2000), or intentionally not followed due to disagreements about the utility, safety, or acceptability of the recommendations (Nadler et al., 2014). Interventions to address quality gaps in perioperative care will likely fail if they do not target the underlying reason for missed recommendations.

Previous quality improvement work in our centre has found that at least 13% of the recommendations made during preoperative consultation are not followed by surgical teams [8]. We undertook a sequential, explanatory mixed-methods study to characterize the types of recommendations that are not followed and to understand barriers and facilitators to implementing these recommendations, with the aim of developing interventions to improve the quality of perioperative care in our setting.

## Methods

The study protocol was approved by our institutional ethics review board. Informed consent was obtained from all participants. This manuscript follows the Good Reporting of a Mixed Methods Study guidelines (O'cathain et al., 2008) and the Strengthening the Reporting of Observational Studies in Epidemiology guidelines for cross-sectional studies when reporting the methods and results for the quantitative stream (von Elm et al., 2007) and the COnsolidated Criteria for REporting Qualitative Research reporting guidelines for reporting the methods and results of the qualitative stream (Tong et al., 2007) (Additional file 1: Appendices 1–3).

### Study design

This sequential, explanatory, mixed-methods study used both the participants selection model and a follow-up explanations model to first characterize and then explain missed recommendations (Creswell, 2017). The quantitative phase was a cross-sectional chart audit to characterize the types and numbers of recommendations made by internists that were not followed by the surgical team (“missed recommendations”) (Ivankova, 2006). This was followed by a qualitative phase of semi-structured interviews with members of the surgical team to understand why recommendations may not have been followed (Ivankova, 2006). The quantitative phase informed the types of surgeons who were recruited for the qualitative phase (e.g., those surgical teams with the greatest number of missed recommendations), the medical topic areas for focus in the interviews (e.g., those medical topics with the most missed recommendations), and guided question development (Creswell, 2017). These variations of the sequential explanatory mixed-methods design use a qualitative strand to explore findings of quantitative results and thus better describe missed perioperative recommendations than either method alone (Creswell, 2017).

### Setting

The Foothills Medical Centre is a quaternary care hospital in Calgary, Alberta, Canada, serving a catchment area of 1.5 million people. The preadmission clinic (PAC) at the Foothills Medical Centre sees 250 to 300 adult patients undergoing elective, semi-urgent, or urgent surgery each month. Patients are referred by their surgeon or are captured through a screening process by booking clerks based on their home medications or the type of surgery. Patients are typically seen between 4 weeks and 1 day before surgery by an internist with the intention of identifying potential medical complications of surgery, providing recommendations for perioperative medication management and other relevant perioperative medical advice. Select patients may also be seen by anesthesiology for pain management or airway considerations.

After the in-person visit, the internal medicine physician dictates a consult note (“PAC visit note”) outlining pertinent medical facts and making recommendations for the patient’s care. The PAC visit note is faxed to the surgeon’s office and uploaded onto the provincial electronic health record, where it can be accessed by any physician in Alberta. The types of recommendations made are not prespecified and are based on the judgement of the internal medicine physician. After surgery, patients are cared for by multidisciplinary teams that may include a nurse practitioner who manages ward issues, residents, fellows, pharmacists, registered nurses, and staff surgeons.

### Quantitative data

A chart audit of all patients assessed in PAC in a 4-week period between April 1 and April 30, 2019 was completed. One reviewer (M.B.) assessed the PAC visit note for the number and type of recommendations using a data extraction template (Additional file 1: Appendix 4). Types of recommendations were prespecified based on common medical topics in perioperative medicine, and included medication, diabetes, respiratory, other endocrine, and cardiac. After surgery, two of three potential reviewers (M.B., S.C., B.M.) assessed the inpatient chart to identify whether the recommendations made in the PAC consult note were followed. Disagreements were resolved by discussion with a third reviewer. A missed recommendation was defined as a recommendation specified in the PAC consult note that was not followed. A completed recommendation was a recommendation made in the PAC consult note by that was followed as outlined.

To calculate the proportion of recommendations followed for categories of recommendations, we considered only consult notes that made the recommendation in the denominator; for example, only patients who were on home medications could be expected to have recommendations related to management of their home medications. We excluded patients who did not have any recommendations, patients with missing data, and patients whose surgeries had not been completed at the time of analysis (3 months after the PAC clinic visit). Chi-squared tests were used to compare categorical data, and the Mann-Whitney *U* test was used to compare non-parametric data. Statistical analysis was completed in Stata (version 11.2, StataCorp, College Station, TX). A *p* value of less than 0.05 was considered significant.

### Qualitative data

Semi-structured, 1-on-1 interviews with key informants were conducted to understand how PAC visit notes are used by the surgical team. The results of the quantitative phase were used to inform the design of the qualitative interview guide, including medical topic areas (e.g., diabetes, anticoagulation, deep vein thrombosis (DVT) prophylaxis) that were common sources of missed recommendations.

An interview guide was developed using the Consolidated Framework for Implementation Research (CFIR) (Damschroder et al., 2009) and the Theoretical Domains Framework (TDF) (Atkins et al., 2017). Combination of the CFIR and TDF can help understand multi-level implementation (Birken et al., 2017). CFIR is a conceptual framework used to understand factors that influence intervention implementation and effectiveness (Keith et al., 2017). CFIR has five domains: intervention characteristics, inner setting, outer setting, characteristics of individuals, and implementation process. In this study, the PAC visit note recommendation represents the intervention, and the CFIR domain intervention characteristics were used to understand how surgical team members interact with and perceive the PAC visit note during their care for surgical patients. The TDF is a framework used to identify determinants of behaviour change in the context of intervention implementation (Atkins et al., 2017). The TDF (version 2) uses fourteen domains: knowledge; skills; social/professional role and identity; beliefs about capabilities; optimism; beliefs about consequences; reinforcement; intentions; goals; memory, attention and decision processes; environmental context and resources; social influences; emotion; and behavioural regulation. The TDF was used to understand how variations in implementation of the PAC visit note recommendations were influenced by both individual behaviours and context factors. Interview guides were pilot tested with two key informants and adapted before use in the entire cohort. Interview guides are available in Additional file 1: Appendices 5–6.

Interviews lasted 30 to 60 min and were conducted in-person by a research associate with experience in interviewing and qualitative methods who does not work with the participants (K.F.). Interviews were audio recorded and transcribed verbatim, followed by manual cleaning to remove identifying information and ensure accuracy before analysis. Interview participants did not review their transcripts.

Interviews were coded using template analysis (Brooks & King, 2014) based on an a priori codebook developed using the CFIR and TDF domains. Coding was completed by two study team members: a content expert (S.M.R.) who works in PAC as an internal medicine physician and the interviewer (K.F.). Template analysis using established conceptual frameworks guides researchers to recognize themes that may be missed in inductive analysis. Additional codes were developed inductively by thematic analysis when the a priori codes were not applicable, creating a final template that was used to code all transcripts. Codes were independently assigned and disagreements in coding were reconciled between the two coders. Each theme was reviewed in its entirety and its valence was categorized as a facilitator, barrier, or neutral to implementing PAC visit note recommendations. The final coding template was used to create a framework that explained missed recommendations from the perspective of the surgical team. Data were managed using nVivo qualitative data analysis software (version 12.3.0).

Key informants were recruited using purposive criterion sampling of staff, trainees, or faculty working in a perioperative medical field at the Foothills Medical Centre in a discipline with a high number of missed recommendations. Surgery residents were recruited through an invitation circulated by chief residents in surgical programs with the highest numbers of missed recommendations, and surgeons from the same disciplines were recruited by convenience sampling based on previously expressed interest in perioperative medicine. Internists who spent the greatest number of weeks working in PAC per year were invited to participate. Participant role is provided except when reporting the discipline of practice may identify individual participants. Sampling continued until no new themes were identified in data analysis in three consecutive interviews (Guest et al., 2006). Participation was voluntary. Nurse practitioners and surgical residents were compensated for their time spent interviewing. Staff surgeons and internists were not reimbursed.

## Results

### Quantitative results

There were 296 patients scheduled for a PAC visit over the 4-week study period. One patient did not attend their appointment, 25 patients had no recommendations made, and 15 surgeries were postponed, cancelled, or unscheduled; therefore, 255 PAC visit notes were included in our analysis (Additional file 1: Appendix 7). The median age of patients was 66 years (IQR 57–72 years) and 161 patients were female (63.1%).

Overall, 55.7% of PAC visit notes (*n*=142) had all recommendations followed. Patients who had missed recommendations were older than patients who had all recommendations followed (median age 67 years (IQR 60–73 years) and 64 years (IQR 52–71 years), respectively; *p*=0.01) (Table 1). General surgery, spine, neurosurgical, and gynecologic procedures had the greatest numbers of patients with missed recommendations (Table 1).

**Table 1:** Characteristics of patients and surgeries included in the analysis

Characteristic	Full cohort *n* (%)	All recommendations followed *n* (%)	Missed recommendations *n* (%)	*p* value^*^
Cohort	255 (100.0)	142 (55.7)	113 (44.3)	
Age (median, IQR)	66 (57–72)	64 (52–71)	67 (60–73)	0.01^†^
Female	161 (63.1)	96 (59.6)	65 (40.4)	0.10^‡^
Male	94 (36.9)	46 (48.9)	48 (51.1)	
Inpatient surgeries	188 (73.7)	188 (54.3)	86 (45.7)	0.16^‡^
Day surgeries	67 (26.3)	40 (59.7)	27 (40.3)	
Surgical discipline				
General surgery	79 (28.8)	45 (57.0)	34 (43.0)	
Spine surgery	50 (18.2)	23 (46.0)	27 (54.0)	
Neurosurgery	32 (11.7)	13 (40.6)	19 (59.4)	
General gynecology	29 (10.6)	19 (65.5)	10 (34.5)	
Gynecology oncology	26 (9.5)	19 (73.1)	7 (26.9)	
Otolaryngology	11 (4.0)	7 (63.6)	4 (36.4)	
Plastic surgery	10 (3.6)	7 (70.0)	3 (30.0)	
Thoracic surgery	6 (2.2)	1 (16.7)	5 (83.3)	
Orthopedics	9 (3.3)	6 (66.7)	3 (33.3)	
Cardiac surgery	1 (0.4)	1 (100.0)	0	
Dentistry	1 (0.4)	0	1 (100.0)	
Urology	1 (0.4)	1 (100.0)	0	

^†^Mann-Whitney *U* test

^‡^Chi-square

Characteristics of recommendations made by internists in the PAC visit note are presented in Table 2. Recommendations that addressed perioperative management of any home medications were most common (*n*=242 of 248 eligible notes, 97.6%). There was no difference in the proportion of recommendations followed in the preoperative period compared to the postoperative period (50.2%, *n*=103/205; compared with 55.0%, *n*=137/249; *p*=0.31).

**Table 2:** Categories of perioperative recommendations made in PAC visit notes, with proportion of recommendations followed, proportion followed preoperatively, and proportion followed postoperatively

Recommendation Type	Eligible consult notes^a^ *n* (%)	Consult notes with any recommendation *n* (%)	All recommendations followed *n* (%)	Consult notes with preoperative recommendations *n* (%)^b^	Preoperative recommendations followed *n* (%)^c^	Consult notes with postoperative recommendations *n* (%)^b^	Postoperative recommendations followed *n* (%)^c^
All types	255	255 (100.0)	142 (55.7)	205 (80.4)	103 (50.2)	249 (97.6)	137 (55.0)
Any home medication	248 (97.3)	242 (97.6)	144 (59.5)	242 (97.6)	90 (37.2)	122 (49.2)	67 (54.9)
Antiplatelet and anticoagulation							
Home medication	101 (39.6)	97 (96.0)	64 (63.3)	96 (99.0)	62 (64.6)	56 (55.4)	28 (50.0)
DVT prophylaxis	192 (75.3)	137 (71.4)	102 (74.5)	n/a	n/a	137 (71.4)	102 (74.5)
Opioids							
Home medication	28 (11.0)	22 (78.6)	18 (81.8)	22 (78.6)	18 (81.8)	4 (14.3)	0
Withdrawal management	28 (11.0)	1 (3.6)	0	n/a	n/a	1 (3.6)	0
Diabetes							
Home medication	72 (28.2)	60 (83.3)	42 (70.0)	60 (83.3)	44 (73.3)	32 (44.4)	29 (90.6)
Hospital management	72 (28.2)	32 (44.4)	26 (81.3)	n/a	n/a	32 (44.4)	26 (81.3)
Cardiac							
Cardiac biomarkers	192 (75.3)	119 (62.0)	82 (68.9)	n/r	n/r	119 (62.0)	82 (68.9)
Home medications	157 (61.6)	141 (89.8)	114 (80.9)	141 (89.8)	114 (80.9)	51 (32.5)	36 (70.6)
Delirium management	49 (19.2)	5 (10.2)	2 (40.0)	n/a	n/a	5 (10.2)	2 (40.0)

^a^Eligible consult notes refer to the number of PAC visit notes that could have included a recommendation in that category, based on the patient’s past medical history or home medications. This serves as the denominator for the proportions reported

^b^Percent of eligible patients in each row category

^c^Percent of recommendations followed over recommendations made

*n/a* not applicable, *n/r* not reported

Of the patients seen, 39.6% (*n*=101) were on home anticoagulation or antiplatelet medications. Recommendations made for preoperative management were followed less than 65% of the time and postoperative recommendations were followed half of the time (64.6% and 50.0%, respectively; *p*=0.08). Over one-quarter of patients were on medications for diabetes and most of these patients had recommendations related to the management of diabetes in the perioperative period (*n*=60 of 72 eligible consult notes, 83.3%). Only 70.0% of recommendations related to perioperative diabetes medications were followed (*n*=42). Recommendations on management of these medications after surgery were made for less than half of patients (*n*=32, 44.4%), though these postoperative recommendations were commonly followed (*n*=29, 90.6%). Nearly three-quarters of patients were eligible for cardiac biomarker screening (referring to preoperative brain natriuretic peptides and/or postoperative troponin measurement) as per the Canadian Cardiovascular Society guidelines (Duceppe et al., 2017) (75.3%, *n*=192). Of these, only 62.0% (*n*=119) had a recommendation for patients to undergo cardiac biomarker testing, while 68.9% of patients with a recommendation had it completed (*n*=82).

Based on these data, the interview guide was adapted to (1) characterize the process by which PAC visit notes are reviewed and recommendations are entered to identify steps in the process where recommendations may be missed and (2) understand why inpatient surgical teams may not follow recommendations, especially those related to perioperative cardiac biomarker surveillance, diabetes, and anticoagulation. These specific questions were intended to address the overall aim of this study, which was to understand the number of and reasons for missed recommendations in PAC visit notes. This will allow us to better inform interventions to improve the quality of perioperative care in our centre.

### Qualitative data

We interviewed 18 participants, including 2 nurse practitioners (11%), 8 surgical residents (44%), 6 surgeons (33%), and 2 internists (11%) (Table 3). Participants represented neurosurgery (*n*=4, 22%), gynecology (*n*=4, 22%), general surgery (*n*=3, 17%), spine surgery (*n*=2, 11%), orthopedic surgery (*n*=1, 6%), otolaryngology (*n*=1, 56%), and thoracic surgery (*n*=1, 6%). One internist and one surgeon declined to participate due to schedule conflicts.

**Table 3:** Characteristics of interview participants

Characteristic	Number (%)
Faculty status	
Nurse practitioners	2 (11)
Residents	8 (44)
Staff physicians	8 (44)
Discipline of practice	
General surgery	3 (17)
Orthopedic surgery	1 (6)
Neurosurgery	4 (22)
ENT surgery	1(6)
Gynecology	4 (22)
Spine surgery	2 (11)
Thoracic surgery	1 (6)
Internal medicine	2 (11)

*ENT* otolaryngology

We used 31 themes in the final codebook (Additional file 1: Appendix 8). Twenty-five codes were based on constructs from CFIR and TDF and 6 were developed through inductive analysis. We separated social context and networks into two subconstructs (peer pressure and social influences, and external networks and internal networks, respectively) to discriminate between similar but different ideas. The six codes developed inductively captured specific medical topics (for example, anticoagulation, diabetes), critiques or suggestions for improvement of the PAC visit note, and differing ideas of the intention of the PAC visit note. Relevant constructs are listed in brackets in the reporting of results.

**Fig. 1:**
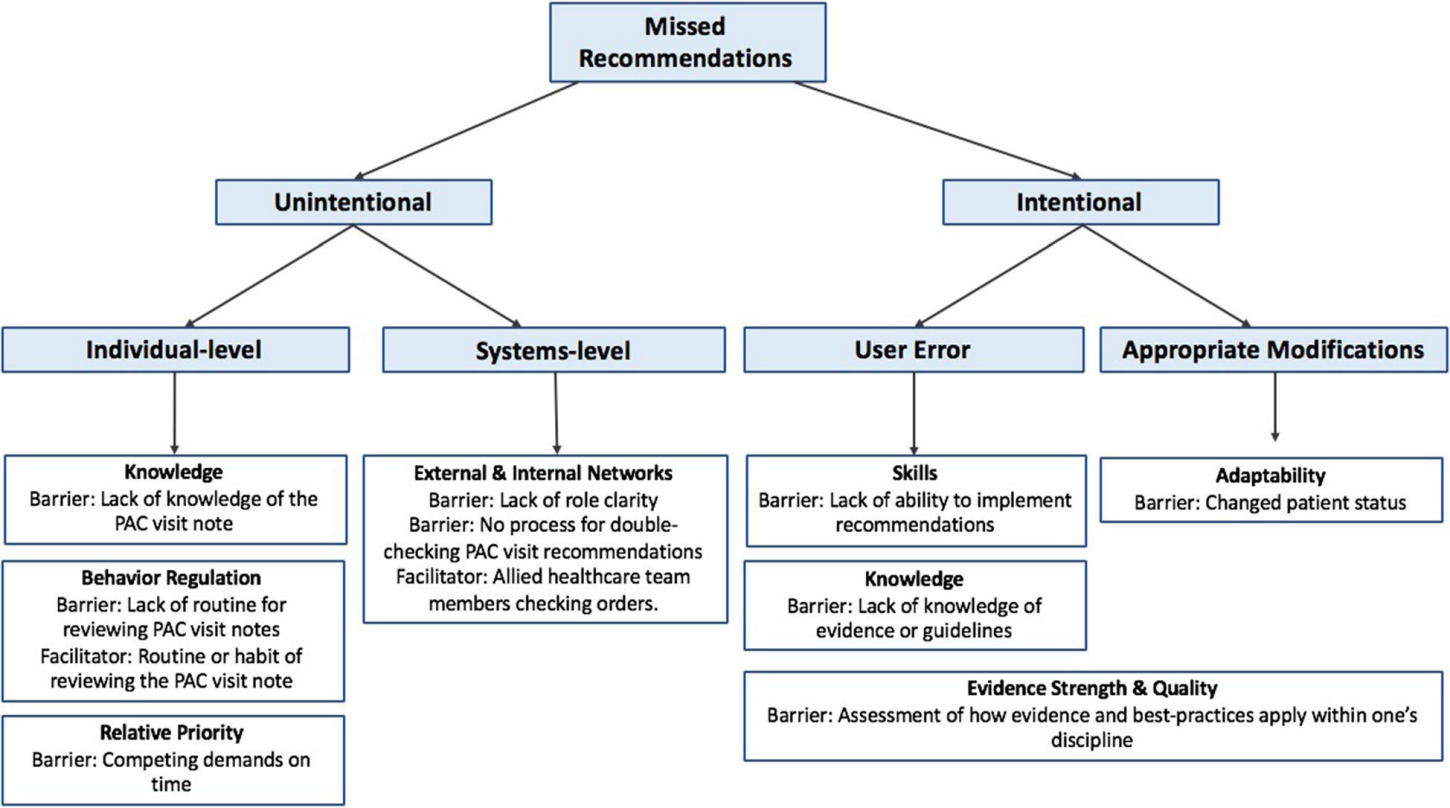
Framework for understanding missed perioperative recommendations, with examples stratified by construct

We categorized reasons for missed recommendations into a conceptual framework which delineates between different kinds of unintentionally and intentionally missed recommendations (Fig. 1). Our qualitative data shows that surgical teams may unintentionally miss recommendations due to both individual-level and systems-level factors. Intentionally missed recommendations can be divided into appropriate modifications (recommendations which surgical teams did not follow because they were not safe or reasonable for their patients) and user error (recommendations which were not followed due to misinterpretation or lack of knowledge of evidence and best practices). Additional supportive quotes are provided in Table 4.

**Table 4:** Variability in the reported and perceived processes for reviewing the PAC visit note by residents, nurse practitioners, and staff surgeons

Driver of missed recommendations	Exemplar quotation
Unintentionally missed recommendations	
Individual-level drivers	
Knowledge of the PAC visit note (Barrier)	“It’s hard to know who was seen by internal medicine before and who was not” (P06, nurse practitioner)
Behaviour regulation (Facilitator)	“Certainly before we’re operating on any patient I look to see if there’s [a PAC visit] note on [our EHR], and so if there is, I read it... it took me a while to make it into a habit where I like will look at it before every patient, to make sure there’s anything I need to do” (P08, surgical resident, ENT)
Behaviour regulation (Barrier)	“It’s just not in [the resident's] routine practice [to look at the PAC visit note], cause not everyone has preop consults… they just have their plan of postop orders, and they just put them in without looking, even for patients that go to the OR from the unit, they just do the same thing all the time” (P06, nurse practitioner)
Relative priority (Barrier)	“When we finish a surgery [and] we put in a post-op order, [that's] typically the first sense we’ll get to as to how medically complex they are. Having said that, when you have time in the evenings, like at the Sunday before your week it is nice to be able to sit down and look through the cases that you have coming for the week, but that takes time and is not always something that’s realistic” (P04, surgical resident, neurosurgery)
Systems-level drivers	
External and internal networks (Barrier)	“No, I mean not any more than any other service where there’s fellows and senior residents and juniors residents and everyone’s is part of the team, and everyone’s trying to do what’s best for the patient, so we’re all looking at things, we’re all trying to you know help each other out, but there’s no, I don’t think there’s a formal process unless the chief resident has made it a priority for him or herself to go through and say I’m gonna look at every preop assessment and make a point of it, there’s no like safety check” (P12, obstetrics and gynecology) “Not terribly often, no... if I had to double check every drug [or] order a resident put in I wouldn’t get anything else done,” (P18, neurosurgery)
External and internal networks (Facilitator)	“The junior resident puts the orders, but I look up the medicine consult and still review it, ok they asked for troponins, they did this and that, so don’t forget about this and that...then [the fellow] double [checks], he looks at it too” (P01, general surgery resident) “No, there’s not [a mechanism to check if orders are entered], if it’s a medication we have a pharmacist on our unit... I would say she’s like a safety net” (P17, gynecologic oncology staff)
Intentionally missed recommendations	
User error	
Skills (Barrier)	“I know you’re supposed to do basal bolus insulin... but the concern would be like how do you titrate it, how do you get them off of it to go home, all those things and so, I just use the sliding scale to like bring them down if they’re high and then let them ride it out” (P06, nurse practitioner)
Knowledge of guidelines and evidence (Barrier)	“We are not understanding whether all those [troponin] suggestions apply to all patients and then who should act [on abnormal results]... [implementation] initially was a little bit vague... I can’t do this so I’m gonna ignore it, and that’s maybe not the best thing for people overall” (P14, staff spine surgery)
Appropriate modifications	
Adaptability (Barrier)	“A lot of times their recommendations tend to be to prevent that from happening, so by that point the cat’s kind of out of the bag in terms of what they thought when the patient was well two weeks prior to surgery... maybe [their recommendation] doesn’t apply anymore, we need a more updated plan” (P04, neurosurgery resident).
Unclassifiable	
Evidence strength and quality	“Things like Xarelto, these other blood thinners, I tell patients different instructions than internal medicine simply based on experience, not evidence” (P14, neurosurgery staff)

### Unintentional missed recommendations

Unintentional missed recommendations occurred when participants were unaware of PAC visit recommendations because they did not read the PAC visit note. Many participants expressed surprise at the proportion of recommendations that were not followed by surgical teams.

#### Individual-level drivers of missed recommendations

Participants from all groups reported that the most junior person on the surgical team was responsible for reading the PAC visit note and entering relevant orders after the end of the surgical case; this was the resident in most instances (Social Identity and Role).

We found that surgical team members were often unaware that patients may have had a PAC visit note (Knowledge) (Fig. 1). One neurosurgery resident stated “It wasn’t right away in my training [that I was told about the PAC visit note], it wasn’t something that was explicitly told to us, that, to look for when you started putting post-op orders... I wouldn’t look, and I didn’t know they were there [until] probably mid-way through first year” (P04).

There was variability between residents regarding whether they had an established routine or process for reading the PAC visit note (Behaviour Regulation) (Fig. 1). The absence of an established routine acted as a barrier to implementing recommendations while an established routine was a facilitator of implementing recommendations. The process of reviewing all surgical patients for a PAC visit note was able to overcome a lack of knowledge of which patients had been in PAC. For example, one surgical resident stated “Pretty much for all of our [elective] patients, I’ll ... see if they’ve been seen [by PAC internists], and if so then I typically try and implement most of the suggestions that they’ve recommended... I actually usually look at them either the night before or the morning of” (P10). Some surgical team members reviewed the PAC visit note only if they had time, while other residents always reviewed the PAC visit note (Relative Priority). Overall, this suggests that a routine of always reviewing the PAC visit note was the most important individual-level driver of missed PAC recommendations.

#### Systems-level drivers of missed recommendations

Similar to individual processes to view PAC visit notes and enter recommendations, surgical team members reported inconsistent systems-level mechanisms to ensure that the PAC visit note had been read and recommendations had been followed (External and Internal Networks) (Fig. 1). Team-based processes where orders were consistently reviewed to ensure all recommendations had been ordered were considered a facilitator of implementing PAC recommendations, and when this process was undefined, it was considered a barrier.

Some surgical teams relied on allied healthcare team members, including charge nurses and pharmacists, to notice omissions in the orders. As one neurosurgery resident stated, “No [one is checking orders], not from the surgical team perspective... the orders [are] put in after surgery... and most charge nurses will review and say GIM said to start this medication after 48 hours” (P04). Other surgical teams had a defined chain of responsibility that involved only physicians, where the more senior residents were double-checking the work of junior residents who entered orders.

While some staff surgeons reported reviewing the PAC visit note, none endorsed checking the postoperative orders to ensure that PAC recommendations had been followed and many stated that they never checked postoperative orders entered by residents. One staff neurosurgeon stated, “If I had to double check every drug [or] order a resident put in, I wouldn't get anything else done” (P18). Contrary to resident self-report, staff surgeons believed that the residents were consistently using the PAC visit note to guide postoperative order entry. This suggests a disconnect between the reported behaviours of surgical residents and the perceptions of staff surgeons, which may account for some unintentionally missed PAC visit note recommendations.

### Drivers of intentional missed recommendations

We classified intentional missed recommendations into user error, referring to the decision to not follow a recommendation based on a mistake in judgement, or appropriate modifications, when surgical teams chose not to follow a recommendation because of changing patient status (Fig. 1). In addition, we observed that themes that explained the missed recommendation clustered by the medical topic of the recommendation.

#### User error

Participants did not follow postoperative diabetes recommendations because of lack of skills to manage and titrate insulin, even when recommendations were specific about dosages and regimens (Skills) (Fig. 1). Residents felt that “we’re so not used [to prescribing] insulin, we’re worried [about] giving little or too much, and then we don’t really have the time or the capacity to make some fine adjustments... and patients are further more complicated, their oral intake is all messed up, so [diabetes] is... the thing that we’re struggling the most with” (P01, general surgery resident). Participants relied on sliding scale insulin to get patients through the postoperative period due to increased comfort with this regimen compared to basal bolus insulin therapy, even when there were PAC visit note recommendations to use basal bolus insulin therapy.

Members of the surgical team did not always follow recommendations for postoperative troponin surveillance because they did not understand the rationale of testing or benefits to patients (Knowledge) (Fig. 1). For example, “Some people don’t feel that troponin monitoring and the interventions actually result in changes to patient’s [management]... so even when we monitor them and [the troponin] go[es up] by five points, it’s... not like it’s a real problem” (P06, nurse practitioner).

#### Appropriate modifications of recommendations

Internists reported difficulty providing postoperative recommendations for patients because of anticipated physiologic changes after surgery and the difficulties in anticipating all the possible complications (Adaptability) (Fig. 1). One internist stated “It’s not realistic [to provide postoperative recommendations]... if they need help after the surgery, they should call for help... but to guess all the pathways that the patient could go down from what the problems may or may not be, what the surgeons may or may not be comfortable with... there are too many variables’ (P15, internist). Surgical residents agreed, stating ‘from our standpoint, our postop patients are not... the same person they were three days before surgery” (P12, gynecologic oncology resident).

#### Unclassifiable

Intentionally missed recommendations related to perioperative anticoagulation management were intentionally not followed when surgical team members did not agree with the evidence-base for the recommendations (Evidence Strength and Quality) (Fig. 1). This occurred when surgical team members reported that the guidelines used by internists to make recommendation did not apply to their specific surgical discipline based on their evaluation of the supporting evidence. In these cases, it is not possible to make a statement about which course of action is safest for patients.

## Discussion

This sequential, explanatory mixed-methods study identified that nearly half of all recommendations made in the PAC visit note were not followed by surgical teams using chart audit methodology. Postoperative anticoagulation management recommendations were most commonly missed. Semi-structured interviews with surgical team members identified that recommendations were unintentionally missed when surgical team members did not have a routine or habit for reviewing PAC visit note recommendations. Drivers for intentionally missed recommendations varied by medical topic and occurred when surgical team members did not have the skills to implement recommendations (diabetes), did not understand the rationale for the recommendations (postoperative troponin surveillance), or did not agree with the evidence based of the recommendation (anticoagulation). Together, these results can inform quality improvement for perioperative medical management and implementation of perioperative medical teams in other centres.

Previous qualitative work has suggested that surgeon preferences and healthcare team beliefs may act as barriers to implementation of Enhanced Recovery After Surgery best practices (Nadler et al., 2014). Further, the barriers to guideline adherence by physicians include skepticism, low skills, and low knowledge of guidelines (Cabana et al., 1999). We similarly identified systems-level and individual-level drivers of unintentionally missed recommendations, such as lack of knowledge of evidence or best practices, lack of awareness of the preoperative consultation, and lack of role clarity among members of the surgical team. Our data also suggests that some missed recommendations are intentional; for example, some missed recommendations occurred due to low skills for management of medical issues or skepticism of evidence and guidelines.

Interventions to improve uptake of perioperative recommendations should therefore not focus solely on knowledge-sharing with surgical teams, but should also address systems-level barriers such as order-entry processes, role clarification, and skills. These interventions should target all members of the surgical team rather than just physicians or trainees. Due to the number of missed recommendations due to appropriate modifications, investigators studying missed perioperative recommendations should also consider that there may always be a background rate of missed recommendations.

Further, these results may inform evaluation of the effectiveness of perioperative consultations. Despite decades of evaluation, it is not clear if these consultations improve the safety or quality of surgical care to justify the increased cost to health systems or burden on patients and their caregivers. Medical guidelines often make conflicting recommendations on perioperative management (Duceppe et al., 2017; Ruzycki et al., 2020) and substantial variation in patient selection, clinical pathways, and models of care for preoperative medical consultation have been reported [25, 26]. For example, some studies have reported no improvement in the quality of care received in patients who had a perioperative consult (Auerbach et al., 2007). However, these authors did not evaluate whether the recommendations made by the perioperative consult were actually implemented by the surgical team, which is a common limitation of studies that evaluate the effectiveness of perioperative medical consultations. Our results demonstrate that variable implementation of medical recommendations may explain the discrepant results of studies evaluating perioperative consultations. Further, our results suggest that implementation of recommendations should be measured and optimized before perioperative programs are evaluated, to ensure that results reflect the effect of the consultation rather than the implementation of recommendations. Investigators may use frameworks such as that described by Proctor et al. to evaluate implementation (Proctor et al., 2011).

Understanding these barriers and facilitators has led to recommendations to improve the quality of perioperative care in our setting. First, surgical residents should be taught about the PAC visit note during their orientation to residency. This orientation should also enforce that staff surgeons expect that the residents are implementing the PAC visit note recommendations. Second, surgical teams should formally clarify who is responsible for reviewing the PAC visit note and who is responsible for verifying that recommendations are ordered correctly. Third, surgical training programs should consider incorporating perioperative medical topics such as diabetes into their academic curriculums, to build skills in management of these issues. Lastly, internal medicine physicians should work with surgeons to create discipline-specific anticoagulation protocols that are acceptable to all stakeholders.

There is considerable variation in perioperative care delivery across centres (Flaker et al., 2016; Rohatgi et al., 2016) which may limit the transferability the results of our single-centre study. Systems-level factors like the availability of electronic health records and integration of inpatient and outpatient services may influence the proportion of unintentionally missed recommendations in other settings. In addition, our interview participants are likely to be more interested in perioperative medicine than non-participants. This may have increased the number of participants who are aware of the PAC visit notes and biased our results towards intentional missed recommendations. Lastly, we did not examine the difference in clinical outcomes between patients who had followed or missed recommendations due to small sample size. This should be examined in a follow-up study.

## Conclusions

Overall, the results of this study aid research into the effectiveness of perioperative medical consultation by emphasizing the need to evaluate implementation of recommendations concurrently with other outcomes. In addition, these results provide a framework for understanding missed recommendations as unintentional and intentional, which allows for the design of interventions that target the barriers and facilitators of following recommendations in other settings.

## Supplementary Information

**Additional file 1:** Appendix 1 – 8.

## Data Availability

Data is available up on reasonable request from the corresponding author. Qualitative data will be de-identified prior to sharing.

## Abbreviations

PAC: Preadmission clinic
TDF: Theoretical Domains Framework
CFIR: Consolidated Framework for Implementation Research
