# Supplementary material for: Barriers and facilitators of following perioperative internal medicine recommendations by surgical teams: a sequential, explanatory mixed-methods study
Source: Perioper Med (Lond). 2022 Feb 1;11:2. doi: 10.1186/s13741-021-00236-x (PMC8805252; doi:10.1186/s13741-021-00236-x)
Supplement: Supplementary file 1 — Additional file 1: Appendix 1 – 8. [file 13741_2021_236_MOESM1_ESM.docx]

**Appendices**

| 1. STROBE Checklist for cross-sectional studies.................................................................... | 2 |
| --- | --- |
| 2. COREQ Checklist for qualitative studies............................................................................ | 4 |
| 3. GRAMM Checklist for mixed methods studies.................................................................. | 7 |
| 4. Data Extraction Template for Chart Audit......................................................................... | 8 |
| 5. Interview Guide (Surgical team members)........................................................................ | 9 |
| 6. Interview Guide (Staff internists)...................................................................................... | 12 |
| 7. Patient Flow Chart............................................................................................................. | 14 |
| 8. Final Codebook.................................................................................................................. | 15 |

**Appendix 1:** **STROBE Statement**—Checklist of items that should be included in reports of ***cross-sectional studies***

|  | Item No | Recommendation | Page No |
| --- | --- | --- | --- |
| **Title and abstract** | 1 | (*a*) Indicate the study’s design with a commonly used term in the title or the abstract | n/a |
|  |  | (*b*) Provide in the abstract an informative and balanced summary of what was done and what was found | 2 |
| Introduction | | | |
| Background/rationale | 2 | Explain the scientific background and rationale for the investigation being reported | 4 |
| Objectives | 3 | State specific objectives, including any prespecified hypotheses | 4-5 |
| Methods | | | |
| Study design | 4 | Present key elements of study design early in the paper | 5 |
| Setting | 5 | Describe the setting, locations, and relevant dates, including periods of recruitment, exposure, follow-up, and data collection | 6 |
| Participants | 6 | (*a*) Give the eligibility criteria, and the sources and methods of selection of participants | 6 |
| Variables | 7 | Clearly define all outcomes, exposures, predictors, potential confounders, and effect modifiers. Give diagnostic criteria, if applicable | 6-7 |
| Data sources/ measurement | 8* | For each variable of interest, give sources of data and details of methods of assessment (measurement). Describe comparability of assessment methods if there is more than one group | 6-7 |
| Bias | 9 | Describe any efforts to address potential sources of bias | 7 |
| Study size | 10 | Explain how the study size was arrived at | 6 |
| Quantitative variables | 11 | Explain how quantitative variables were handled in the analyses. If applicable, describe which groupings were chosen and why | 7 |
| Statistical methods | 12 | (*a*) Describe all statistical methods, including those used to control for confounding | 7 |
|  |  | (*b*) Describe any methods used to examine subgroups and interactions | n/a |
|  |  | (*c*) Explain how missing data were addressed | n/a |
|  |  | (*d*) If applicable, describe analytical methods taking account of sampling strategy | n/a |
|  |  | (*e*) Describe any sensitivity analyses | n/a |
| Results | | | |
| Participants | 13* | (a) Report numbers of individuals at each stage of study—eg numbers potentially eligible, examined for eligibility, confirmed eligible, included in the study, completing follow-up, and analysed | 10 |
|  |  | (b) Give reasons for non-participation at each stage | 10 |
|  |  | (c) Consider use of a flow diagram | Appendix |
| Descriptive data | 14* | (a) Give characteristics of study participants (eg demographic, clinical, social) and information on exposures and potential confounders | Table 1 |
|  |  | (b) Indicate number of participants with missing data for each variable of interest | n/a |
| Outcome data | 15* | Report numbers of outcome events or summary measures | Table 1 |
| Main results | 16 | (*a*) Give unadjusted estimates and, if applicable, confounder-adjusted estimates and their precision (eg, 95% confidence interval). Make clear which confounders were adjusted for and why they were included | n/a |
|  |  | (*b*) Report category boundaries when continuous variables were categorized | n/a |
|  |  | (*c*) If relevant, consider translating estimates of relative risk into absolute risk for a meaningful time period | n/a |
| Other analyses | 17 | Report other analyses done—eg analyses of subgroups and interactions, and sensitivity analyses | 11-19 |
| Discussion | | | |
| Key results | 18 | Summarise key results with reference to study objectives | 19-20 |
| Limitations | 19 | Discuss limitations of the study, taking into account sources of potential bias or imprecision. Discuss both direction and magnitude of any potential bias | 22 |
| Interpretation | 20 | Give a cautious overall interpretation of results considering objectives, limitations, multiplicity of analyses, results from similar studies, and other relevant evidence | 22 |
| Generalisability | 21 | Discuss the generalisability (external validity) of the study results | 22 |
| Other information | | | |
| Funding | 22 | Give the source of funding and the role of the funders for the present study and, if applicable, for the original study on which the present article is based | 3 |

**Appendix 2:**

Consolidated criteria for reporting qualitative studies (COREQ): 32-item checklist

| **No** | **Item** | **Page Number** |
| --- | --- | --- |
| **Domain 1: Research team and reflexivity** |  |  |
| Personal Characteristics |  |  |
| 1. | Interviewer/facilitator | 8 |
| 2. | Credentials | 1 |
| 3. | Occupation | 8 |
| 4. | Gender | n/r |
| 5. | Experience and training | 8 |
| Relationship with participants |  |  |
| 6. | Relationship established | 8 |
| 7. | Participant knowledge of the interviewer | 8 |
| 8. | Interviewer characteristics | 8 |
| **Domain 2: study design** |  |  |
| Theoretical framework |  |  |
| 9. | Methodological orientation and Theory | 9 |
| Participant selection |  |  |
| 10. | Sampling | 9 |
| 11. | Method of approach | 9 |
| 12. | Sample size | 14 |
| 13. | Non-participation | 14 |
| Setting |  |  |
| 14. | Setting of data collection | 8 |
| 15. | Presence of non-participants | 8 |
| 16. | Description of sample | 14 |
| Data collection |  |  |
| 17. | Interview guide | 8 |
| 18. | Repeat interviews | n/a |
| 19. | Audio/visual recording | 8 |
| 20. | Field notes | n/r |
| 21. | Duration | 8 |
| 22. | Data saturation | 9 |
| 23. | Transcripts returned | 9 |
| **Domain 3: analysis and findings** |  |  |
| Data analysis |  |  |
| 24. | Number of data coders | 9 |
| 25. | Description of the coding tree | 8-9, Appendices 2 & 3 |
| 26. | Derivation of themes | 9 |
| 27. | Software | 9 |
| 28. | Participant checking | n/r |
| Reporting |  |  |
| 29. | Quotations presented | Table 4 |
| 30. | Data and findings consistent | 15-19 |
| 31. | Clarity of major themes | 15-19 |
| 32. | Clarity of minor themes | n/r |

|  |  |
| --- | --- |

**Appendix 3.** Good Reporting of A Mixed Methods Study (GRAMMS)

(1) Describe the justification for using a mixed methods approach to the research question – page 5

(2) Describe the design in terms of the purpose, priority and sequence of methods – page 5

(3) Describe each method in terms of sampling, data collection and analysis – pages 5-9

(4) Describe where integration has occurred, how it has occurred and who has participated in it – page 5

(5) Describe any limitation of one method associated with the presence of the other method – page 21

(6) Describe any insights gained from mixing or integrating methods – pages 19-20, Figure 1

**Appendix 4:** **Data Extraction Template for Chart Audit**

| **Patient Demographics** | | | | | | | | |
| --- | --- | --- | --- | --- | --- | --- | --- | --- |
| Age |  | | | | | | | |
| Sex |  | | | | | | | |
| Surgery type |  | | | | | | | |
| Surgery date |  | | | | | | | |
| **Recommendations** | | | | | | | | |
|  | Preoperative Recommendations | | | | Postoperative Recommendations | | | |
|  | Eligible? | Present | Followed? | None | Eligible? | Present | Followed? | None |
| Any home medication |  |  |  |  |  |  |  |  |
| Antiplatelet/anticoagulation medications |  |  |  |  |  |  |  |  |
| DVT prophylaxis |  | | | |  |  |  |  |
| Opioid medications |  |  |  |  |  |  |  |  |
| Opioid withdrawal |  | | | |  |  |  |  |
| Diabetes medication |  |  |  |  |  |  |  |  |
| Diabetes (non-medication) |  |  |  |  |  |  |  |  |
| Other endocrine |  |  |  |  |  |  |  |  |
| Cardiac biomarkers |  |  |  |  |  |  |  |  |
| Cardiac medications |  |  |  |  |  |  |  |  |
| Delirium management |  | | | |  |  |  |  |
| Respiratory management |  | | | |  |  |  |  |
| **Total** |  |  |  |  |  |  |  |  |

**Appendix 5: PAC Note Interview Guide for Surgical Team Members – Feb 12, 2020**

| **Suggested Domain^*^** | **Question(s)** |
| --- | --- |
| n/a | Tell us a little bit about yourself! Your role, your year, experience, specialties? |
| Knowledge | How often do you encounter the PAC note?  When/how were you first introduced to it? By whom/what setting?  (*Prompt: Curriculum, attending, other resident, what context*) |
| Behaviour Regulation, Available Resources | Can you describe your workflow when planning a surgery for me?  *(Prompt patient comorbidities, post-op care, entering medications*)  *(Medical planning; meeting patient; med recs, EHR/chart, etc)* |
| Knowledge | How do you know which patients have Internal Medicine recommendations?  *(Prompt: Is it clear why you would or would not send a person to PAC?)* |
| Environmental Context and Resources, Behaviour Regulation, Relative Priority | At what points are you accessing or referring to the PAC note? |
| Social/Professional Role & Identity, Behavioural Regulation, Environmental Context, Reinforcement | Whose job is it to enter postoperative orders?  Who looks at and orders the contents of PAC recommendations?  Who reviews the postoperative orders?  Is the staff surgeon going over your work or double checking (*Residents)*?  Do you review the residents' postoperative orders (*Surgeons, NPs*)?  Whose job do you think it should be? Is it the surgeon’s job to review this work? |
| Memory, attention and decision processes, Behavioural Regulation | Is there a process to ensure that the recommendations are implemented?  Do you double check? When would you return to the note? |
| Environmental Context | In what forms do you review the PAC note? *(paper chart, netcare, etc?)* |
| Memory, attention and decision processes | Do you ever review the PAC note again after entering order?  (*Prompt: If there a patient has symptoms (chest pain, blood sugar?). An unrecognized medication? An emergency?*) |
| Intervention Characteristics, Relative Priority, Belief about Consequences | How valuable are the contents of the note to you? |
| Belief about Consequences | Is it important to implement recommendations completely?  What factors might change how you would implement recommendations? |
| Behavioural Regulation, Relative Priority | What is your process of going over the note?  Are there areas that are more/less helpful?  *(Prompt: past medical history, medications, review of systems, physical exam, investigations, impression, and plan.)* |
| Evidence Strength & Quality, Beliefs about Consequences, Knowledge | Do you have any thoughts about the evidence base the recommendations draw on? *(Prompt: regarding diabetes management, anticoagulant management?*)  Do they differ from recommendations in your field/specialty?  *(Prompt: Are you aware of evidence or recommendations in your specialty?)*  How do you navigate the differences? |
| Adaptability | Are recommendations made by internists in preadmission clinic notes adaptable for changing patient status? |
| Intervention Source | Do you think internists who work in PAC understand enough about surgery to make useful recommendations for management of complex medical patients before and after surgery? |
| Complexity | Are the recommendations realistic? Have you seen examples of unrealistic recommendations? |
| Design Quality and Packaging | Are the recommendations easy to find and understand? To implement? |
| Available Resources | Do you have enough time/energy to read and implement the PAC notes? |
| Complexity, Design Quality and Packaging | Are notes too short or too long? |
| Suggestions | Other strengths and weaknesses of the note? Any suggestions or requests? |
| Belief about Capabilities, Social/Professional Role & Identity | Should surgeons be able to manage patients with comorbidities/ complex medical illnesses w/o help from internists?  *(Prompts: managing diabetes? Deciding about anticoagulation?)* |
| Skills, Beliefs about Capabilities | How confident would you be medically managing a patient without a PAC visit note?  Are there specific areas that you do or do not need help or recommendations?  *(Prompt: diabetes, anticoagulation, unfamiliar medications)* |
| Social Influences | How do you think your peers use and view the note?  Is there a common expectation about how the note will be read and implemented?  Do you feel any pressure to implement the PAC recommendations because they were made by a colleague?  Would you say there is a common opinion or feeling about the PAC note system amongst your colleagues? |

^*^Questions were mapped to domains of the Theoretical Domains Framework and the Consolidated Framework for Implementation Research during development of the interview guide but participant responses could be coded to additional or alternate domains at the discretion of the coders.

**Appendix 6: PAC Note Internist Interview Guide – Feb 27, 2020**

| **Suggested Domain^*^** | **Question(s)** |
| --- | --- |
| n/a | Tell us a little bit about yourself! Your role, experience, specialties? |
| Design Quality and Packaging, Behavioural Regulation | Can you describe your workflow when dictating the PAC note for me?  How do you your plan the note?  Do you have certain things you focus on for before surgery in most patients?  Do you have certain things you focus on for after surgery in most patients? |
| Social/Professional Role & Identity | Who is the intended audience for your note?  *(Prompt: who reads it? Are there multiple people?)*  Who looks at/records/orders contents of PAC recommendations?  Who is usually responsible for implementing or not implementing the recommendations that you make in a PAC note?  Who is most responsible for implementing or not implementing the recommendations that you make in a PAC note? |
| Behavioural Regulation, Memory, attention, and decision process | Is there any process in Internal Medicine or Surgery to ensure that the recommendations are implemented?  If you recommend something in the PAC note that you feel is very important, do you check to ensure that it is implemented?  Do you think the surgical team has a process to ensure recommendations are followed? |
| Design Quality and Packaging, Beliefs about Consequences | What aspects of the PAC note do you feel are most helpful?  (*Prompt: to patients, to surgeons, to family doctors*)  Are there areas that are more or less helpful?  (*Prompt:* *past medical history, medications, review of systems, physical exam, investigations, impression, and plan*)  Are there medical comorbidities that are more/less important to provide recommendations on?  (*Prompt: diabetes, anti-coagulation, DVT prophylaxis, troponins*) |
| Evidence Strength & Quality | Have you encountered differences in the evidence base or guidelines between internal medicine and surgery?  *(Prompt*: *diabetes management, anticoagulant management, troponins?)*  Do you try to highlight guidelines as part of your recommendations?  (*Prompt: anti-coagulation management, for diabetes, for troponins)*  Have you ever encountered resistance or pushback on your recommendations from the surgical team? |
| Adaptability | Is it possible or realistic to provide recommendations for management of patients after surgery?  Are recommendations made by internists in preadmission clinic notes adaptable for changing patient status?  Is the post-operative period too complex to make recommendations about diabetes or anti-coagulation management in the PAC note?  Is it possible to provide a range of recommendations that vary by patient scenario, or is it too complex? |
| Beliefs about Consequences | Is it important that the surgical teams implement your recommendations completely?  Are there recommendations that are more important to be recommended than others? |
| Intervention Source | Do you ever feel that your knowledge of surgery and post-operative care limits your ability to make recommendations that are appropriate for post-operative care?  (*Prompt: anti-coagulation management, diabetes, troponins)* |
| Skills, Social/Professional Role & Identity | Does the surgical team know enough about medicine to manage medical issues in the post-operative period?  (*Prompt: anti-coagulation management, diabetes, troponins)*  What medical issues should surgeons be able to manage, and what issues require an internist?  Do surgeons try to manage too much on their own, without help from specialists? |
| n/a | What barriers do you think the surgical team experiences in implementing our recommendations?  Do you have strategies or techniques to reduce these barriers? |
| Social Influences | What would you say the general feeling is about the PAC note system amongst your colleagues?  Is there a common expectation about how the note will be read and implemented? |

**Appendix 7.** Patient flow diagram for inclusion and exclusion in the study.


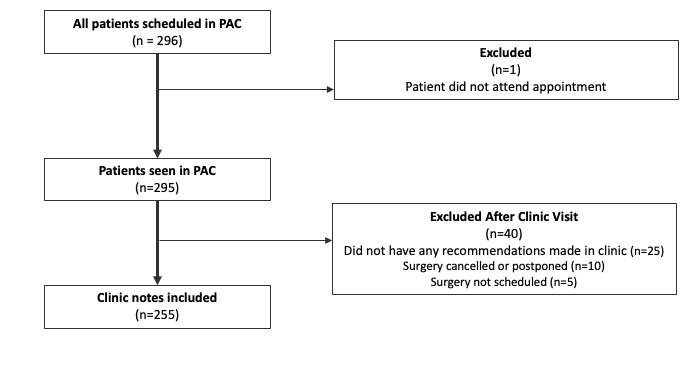


**Appendix 8. Final Codebook.**

|  | **Domain** | | **Definition** | **Framework** |
| --- | --- | --- | --- | --- |
| **1. PAC Note Characteristics** | | |  |  |
|  | Intervention Source | | Perception of key stakeholders about whether the intervention is externally or internally developed. | CFIR |
|  | Evidence Strength & Quality | | Stakeholders’ perceptions of the quality and validity of evidence supporting the belief that the intervention will have desired outcomes. | CFIR |
|  | Value Added (previously: Relative Advantage) | | Stakeholders’ perception of the advantage of implementing the intervention versus an alternative solution. | CFIR |
|  | Adaptability | | The degree to which an intervention can be adapted, tailored, refined, or reinvented to meet local needs. | CFIR |
|  | Complexity | | Perceived difficulty of implementation, reflected by duration, scope, radicalness, disruptiveness, centrality, and intricacy and number of steps required to implement. | CFIR |
|  | Design Quality & Packaging | | Perceived excellence in how the intervention is bundled, presented, and assembled. Ease of use, User Interface (EHR, paper chart, etc) | CFIR |
|  | | | | |
| **2. ENVIRONMENTAL CONTEXT** | | |  |  |
|  | Structural Characteristics | | The social architecture, age, maturity, and size of an organization. |  |
|  | Networks | |  |  |
|  | | External networks | Communication and understanding of relationship between different services at foothills. I.E. Outpatient to Inpatient, Internal med & surgical service, etc | CFIR |
|  | | Internal Networks | Social networks and formal/informal communication within a surgical service or unit at Foothills | CFIR |
|  | Patient Needs & Resources | | The extent to which patient needs, as well as barriers and facilitators to meet those needs, are accurately known and prioritized by the organization. | CFIR |
|  | Relative Priority | | Individuals’ shared perception of the importance of the implementation within the organization. | CFIR |
|  | Available Resources | | The level of resources dedicated for implementation and on-going operations, including money, training, education, physical space, and time. | CFIR |
|  | Access to Knowledge & Information | | Ease of access to digestible information and knowledge about the intervention and how to incorporate it into work tasks. | CFIR |
|  | Reinforcement | | Increasing the probability of a response by arranging a dependent relationship, or contingency, between the response and a given stimulus. Rewards, Incentives, Punishment, Consequents, Reinforcement, Contingencies, Sanctions | TDF |
|  | | | | |
| **3. SOCIAL CONTEXT** | | |  |  |
|  | Social Influences / Culture | | Group identity; Social pressure; Social norms; Group conformity; Social comparisons; Social support; Power; Conflict; Alienation | TDF / CFIR |
|  | Peer Pressure | | Mimetic or competitive pressure to implement an intervention; typically because most or other key peer or competing organizations have already implemented or are in a bid for a competitive edge. | CFIR |
|  | | | | |
| **4. CHARACTERISTICS OF INDIVIDUALS** | | |  |  |
|  | Knowledge | | An awareness of the existence of something. Individuals’ attitudes toward and value placed on the intervention as well as familiarity with facts, truths, and principles related to the intervention. Knowledge (including knowledge of condition/scientific rationale); Procedural knowledge; Knowledge of task environment; Schemas + mindsets + illness representations; Procedural knowledge | CFIR / TDF |
|  | Skills | | An ability or proficiency acquired through practice. Skills, Skills development, Competence, Ability, Interpersonal skills, Practice, Skill assessment | TDF |
|  | Social/professional role and identity | | A coherent set of behaviours and displayed personal qualities of an individual in a social or work setting. Professional identity; Professional role; Social identity; Identity; Professional boundaries; Professional confidence; Group identity; Leadership; Organizational commitment | TDF |
|  | Beliefs about capabilities | | Acceptance of the truth, reality or validity about an ability, talent or facility that a person can put to constructive use. Self-confidence; Perceived competence; Self-efficacy; Beliefs; Self-esteem; Empowerment; Professional confidence | TDF |
|  | Beliefs about consequences | | Acceptance of the truth, reality, or validity about outcomes of a behaviour in a given situation. Beliefs; Outcome expectancies; Characteristics of outcome expectancies; Anticipated regret; Consequents; Appraisal/evaluation/review; Salient events/sensitization/critical incidents | TDF |
|  | Motivation and goals | | Mental representations of outcomes or end states that an individual wants to achieve. Goals (distal/proximal); Goal priority Goal/target setting; Goals (autonomous/controlled); Action planning; Implementation intention; Intention; stability of intention/certainty of intention | TDF |
|  | Memory, attention and decision processes | | The ability to retain information, focus selectively on aspects of the environment and choose between two or more alternatives. Memory; Attention; Attention control; Decision making; Cognitive overload/tiredness | TDF |
|  | Emotion | | A complex reaction pattern, involving experiential, behavioural, and physiological elements, by which the individual attempts to deal with a personally significant matter or event. Fear; Anxiety; Affect; Stress; Depression; Positive/negative affect; Burn-out | TDF |
|  | Behavioural Regulation | | Workflow; anything aimed at managing or changing objectively observed or measured actions; Behaviour that is automatic, conditioned, not consciously recognized, etc. Self-monitoring; Breaking habit; Action planning; Routine/automatic/habit; Direct experience/past behaviour; Representation of tasks; Stages of change model | TDF |
|  |  |  |  |  |
| **5. INDUCTIVE CODES** | | |  |  |
|  | Specific Medical Topics | |  |  |
|  |  | Anti-Coagulation |  |  |
|  |  | Diabetes |  |  |
|  |  | DVT |  |  |
|  |  | Fitness for surgery |  |  |
|  |  | Troponin Monitoring |  |  |
|  | Risk Estimation | | Processes by which risk of various treatment pathways is estimated |  |
|  | Purpose of PAC recommendations (philosophy of note) | | Individual perspectives on the role of the PAC recommendations and how they ought to operate |  |
|  | Communication Pathways | | Ways in which different units or entities within the hospital do or do not communicate; flows of information; communication breakdowns |  |
|  | Suggestions | | Suggestions to improve usefulness of PAC recommendations or recommendation process |  |
|  | Critiques | | Identification of problems with the PAC note recommendations or process |  |
